# Identification of critical residues of influenza neuraminidase in viral particle release

**DOI:** 10.1186/1743-422X-8-14

**Published:** 2011-01-13

**Authors:** Jennifer R Tisoncik, Ying Guo, Katie S Cordero, Jia Yu, Jianwei Wang, Youjia Cao, Lijun Rong

**Affiliations:** 1 Department of Microbiology and Immunology, University of Illinois at Chicago, Chicago, IL 60612, USA; 2 Institute of Materia Medica, Peking Union Medical College and Chinese Academy of Medical Sciences, Beijing 100052, China; 3 College of Life Sciences, Nankai University, Tianjin 300071, China; 4 State Key Laboratory of Molecular Virology and Genetic Engineering, Institute of Pathogen Biology, Chinese Academy of Medical Sciences, Beijing 100052, China

## Abstract

**Background:**

Influenza neuraminidase (NA) is essential for virus release from its host cells and it is one of the targets for structure-based antiviral drug design.

**Results:**

In this report, we established a pseudoviral particle release assay to study NA function, which is based on lentiviral particles pseudotyped with influenza glycoproteins HA and NA as a surrogate system. Through an extensive molecular analysis, we sought to characterize important residues governing NA function. We identified five residues of NA, 234, 241, 257, 286 and 345, four of which (except 345) map away from the active site of NA when projected onto the three-dimensional structure of avian influenza H5N1 NA, and substitutions of these residues adversely affected the NA-mediated viral particle release, suggesting that these residues are critical for NA enzymatic activity.

**Conclusion:**

Through extensive chimeric and mutational analyses, we have identified several residues, which map away from the active site and are critical for NA function. These findings provide new insights into NA-mediated pseudoviral particle release and may have important implications in drug design and therapeutics against influenza infection.

## Background

Influenza virus causes acute respiratory infections resulting in an estimated 300,000 deaths worldwide each year, of which approximately 36,000 deaths occur in the United States alone. Equally concerning is the emergence of new viral strains in the human population, including the ongoing H5N1 epizootic and swine-origin H1N1 pandemic[1-8]. While influenza vaccines are available, they must be reformulated annually to control for antigenic drift and shift of the two major envelope glycoproteins, hemagglutinin (HA) and neuraminidase (NA). HA binds *N*-acetyl neuraminic acid (Neu5Ac) mediating virus entry, whereas NA catalyzes Neu5Ac receptor removal facilitating viral particle release. The abundance of Neu5Ac on the cellular surface can impede influenza egress making NA critical for sustained virus infection. NA is one example where an enveloped virus has evolved a mechanism to promote influenza virus release, making optimal influenza virus spread and infection [9,10].

Several other roles have been proposed for NA including (1) clearance of 'decoy' receptors within the respiratory mucin [11], (2) reduction of viral superinfection [12], and (3) enhancement of viral infectivity [13,14]. NA may also enhance viral infectivity by sequestering plasminogen to facilitate activation of HA; however, this function may be virus specific as a recent study involving the 1918 NA does not support this notion [13-15]. It is interesting to note that, in the absence of efficient NA activity, progeny virions aggregate at the cell surface; however, a release-competent mutant lacking the NA active site was the result of decreased HA binding to Neu5Ac receptors. Thus, there appears to exist a balance of NA and HA activities in orchestrating viral particle release [16-18].

NA exists as a tetramer of identical subunits, each monomer containing an active site that is highly conserved across all influenza A and B viruses [19]. In addition to its enzymatic activity, NA has important modifications that have been shown to influence viral infectivity and possibly glycoprotein function [20]. For instance, loss of a glycan at position 146 is known to induce neurovirulence in mice [13,21]. The NA stalk length has also been demonstrated to be important for enhanced pathogenicity of H5N1 virus [22,23]. Outside of the well characterized active site, few have sought to identify and define important residues of NA. Since NA is one of the two major antigenic glycoproteins of influenza virus, and it is a good target of drug therapeutics, we sought to further characterize the role of NA in this study. Through extensive chimeric and mutational analyses, we have identified several residues, which map away from the active site and are critical for NA function in viral particle release. These findings provide new insights into NA-mediated pseudoviral particle release and may have important implications in drug design and therapeutics against influenza infection.

## Results

### Characterization of influenza NA using HIV/HA pseudotype particles

**Figure 1:**
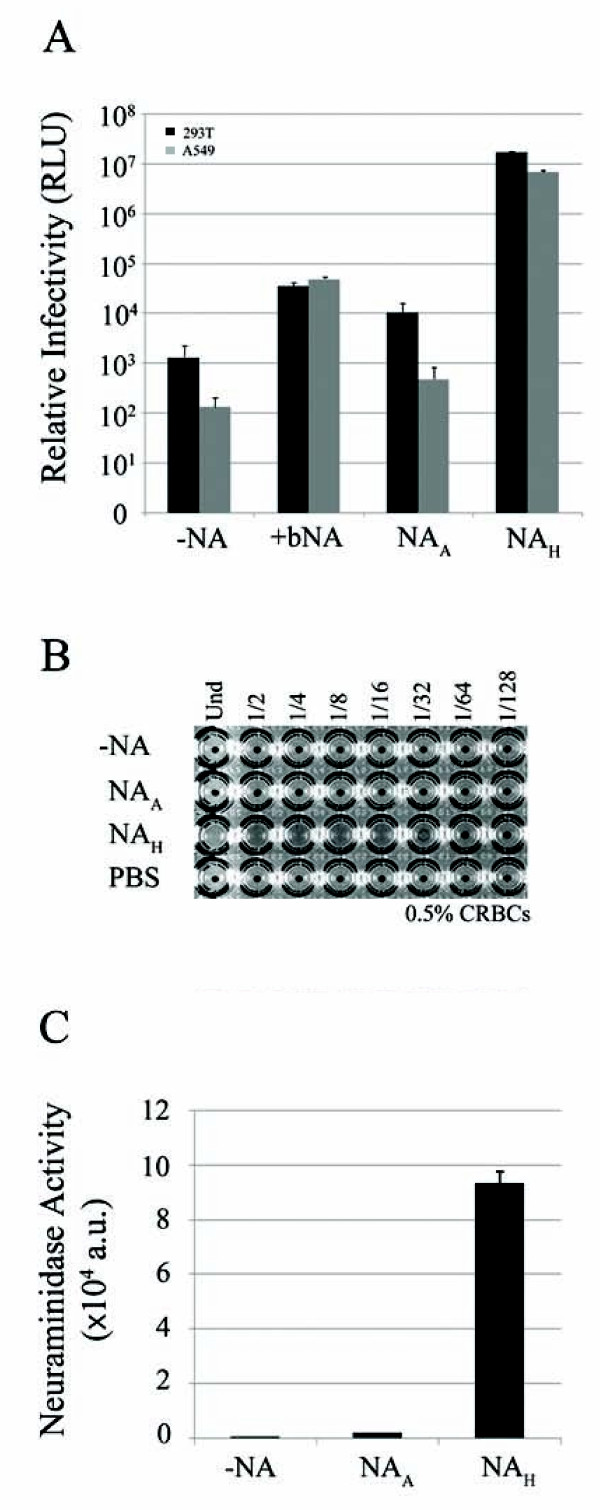
**Characterization of HIV/HA pseudoviral particle release mediated by NA_A _and NA_H_**. *A*, relative infectivity of the pseudovirions determined by luciferase activity (relative light units, RLUs) from infected 293T and A549 target cells. Pseudovirions generated in the absence of NA represent background luciferase levels. As a positive control, HA and HIV plasmids were first introduced into 293T producer cells and the transfected cells were then treated with a commercial neuraminidase twice during pseudovirion production (+bNA). Experiments were performed in triplicates and repeated several times. Error bars indicate standard deviations. *B*, hemagglutination activity from titrated NA_A _and NA_H _pseudovirion populations mixed with chicken red blood cells. PBS serves as a negative control. *C*, neuraminidase activity of NA_A _and NA_H _proteins measured by the release of fluorogenic substrate 4-MUNANA (arbitrary units). Experiments were performed in triplicates and repeated several times, and error bars indicate standard deviations.

Reporter-based HIV/HA pseudotyped viruses were generated by co-transfection of HA and *env*-deficient HIV-1 plasmids into 293T producer cells [24]. The human 293T and A549 target cells were challenged with the producer cell culture supernatants collected 48 h post-transfection and transduction determined by luciferase activity in the target cells. Addition of soluble NA to the culture medium during pseudovirion production enhanced HIV/HA transduction (Figure 1A). Co-transfection of NA from mouse-adapted human virus (PR8), henceforth referred to as NA_H_, with the HA and HIV plasmids resulted in greater transduction efficiency, 1.7 × 10^7 ^and 6.8 × 10^6 ^RLU for 293T and A549 cells, respectively. In contrast, co-transfection of NA from an avian H5N1 virus (NA_A_) resulted in luciferase levels only slightly higher than the background level, 1.3 × 10^3 ^and 1.3 × 10^2 ^RLU for 293T and A549 cells, respectively (Figure 1A).

A hemagglutination assay was used to further explore the discrepancy between NA_H _and NA_A_-mediated pseudovirus production. As shown in Figure 1B, pseudovirions produced in the presence of NA_H _resulted in a hemagglutination titer of 32 HA units/ml, while expression of NA_A _resulted in no hemagglutination, similar to the PBS control. The supernatants derived from co-transfections with HIV vector and NA_H _alone, or with HIV vector, NA_H _and VSV-G, the glycoprotein of vesicular stomatitis virus, resulted in no hemagglutination (results not shown). These results correlate with the luciferase data, further suggesting that NA_A _is deficient in mediating pseudovirion release from the 293T producer cell surface. To determine if the defect was related to its enzyme activity, NA catalysis of fluorogenic substrate (4-MUNANA) was measured. Compared to NA_H_, the avian H5N1 NA appears to be defective in its enzymatic activity (Figure 1C). This may be the result of defective viral genes despite NA_A _being derived from an avian H5N1 virus, or through PCR introduced mutations or both. Nevertheless, we reasoned this defective NA clone could allow us to identify critical residues of NA which had not been identified previously.

### Functional analysis of NA_H_/NA_A _chimeric constructs

**Figure 2:**
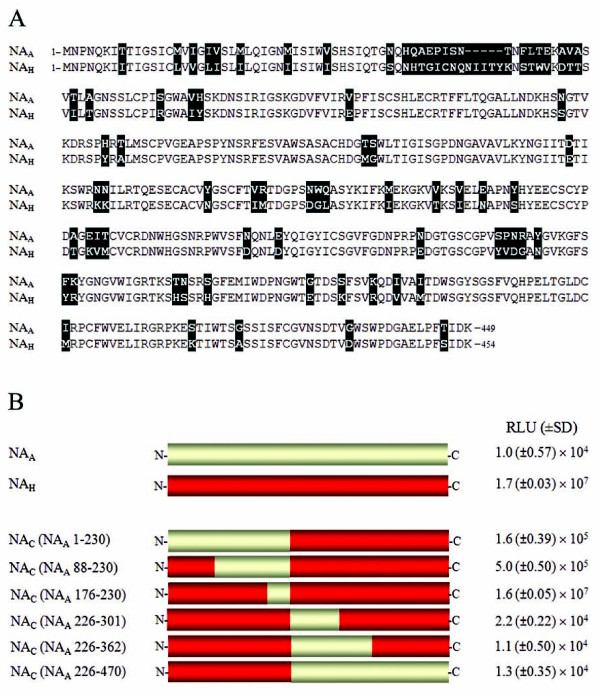
**Alignment of NA_A _and NA_H _amino acid sequences and schematic of NA constructs**. *A*, amino acid sequence alignment of full-length NA_A _and NA_H _proteins. Residue differences are shaded and deletions in the NA_H _linear sequence are denoted by (-) symbol. *B*, schematic of full-length NA_A _and NA_H _proteins and chimeric NA protein panel (NA_C_). NA_H _sequence is colored red and NA_A _sequence is colored yellow. The NA_A _regions represented in NA chimeric proteins are labeled according to N2 numbering. The luciferase activities from infected 293T cells are shown on the right. Values are presented as the average of triplicate samples (± SD).

Comparison of NA_A _and NA_H _primary sequences revealed 83% amino acid identity with a total of 81 amino acid differences (Figure 2A). All the residues previously identified to be important for NA function are identical for these genes with the exception of position 119. Thus, additional residues of NA likely play an important role in NA activity. To delineate the region(s) critical for NA-mediated pseudovirion release, a panel of six chimeric NA constructs was generated and characterized in the luciferase assay (Figure 2B). Chimeric protein NA_C _(NA_A _176-230) behaved like parental NA_H _resulting in 1.6 × 10^7 ^RLU (Figure 2B). Additionally, hemagglutination and NA enzyme activity of NA_C _(NA_A _176-230) was comparable to NA_H _and consistent with the luciferase data (Table 1). Within this region, aa 176-230, there are five variable residues between the two NAs (Figure 2B), and therefore the differences in this region are not important for NA activity. NA chimeric constructs NA_C _(NA_A _1-230) and NA_C _(NA_A _88-230) displayed reduced luciferase levels and enzyme activity, as well as a complete loss of hemagglutination (Figure 2B, and Table 1). Similarly, chimeric proteins NA_C _(NA_A _226-301), NA_C _(NA_A _226-362) and NA_C _(NA_A _226-470) all resulted in a complete loss of NA function. We conclude that variations in the N-terminus of NA_A_, aa 88-176, and the C-terminal portion aa226-301 contribute toward the defective NA_H _phenotype (Figure 2B, Table 1).

**Table 1:** Summary of HIV/HA-mediated relative infectivity and HA and NA activity

NA construct	Luciferase activityª		HA U/ml	4-MUNANA Release (a.u.)
			
	293T cells	A549 cells		
NA_H_	1.7 (±0.03) × 10^7^	6.9 (±0.5) × 10^6^	64	9.3 (±0.41) × 10^4^
E119V	8.1 (±2.10) × 10^5^	1.2 (±0.1) × 10^3^	0	8.3 (±0.51) × 10^3^
N234Y	1.7 (±0.02) × 10^6^	4.3 (±1.00) × 10^5^	16	2.5 (±0.04) × 10^4^
M241R	2.7 (±0.10) × 10^5^	1.6 (±1.1) × 10^2^	0	4.9 (±0.16) × 10^2^
G248W	9.1 (±10.3) × 10^6^	7.5 (±1.7) × 10^5^	32	2.8 (±0.11) × 10^4^
G345R	3.7 (±0.90) × 10^4^	1.3 (±)0.3 × 10^2^	0	4.1 (±0.25) × 10^2^
I257M	1.8 (±0.009) × 10^7^	4.6 (±0.47) × 10^6^	64	8.3 (±0.29) × 10^4^
K286E	1.4 (±0.02) × 10^7^	1.5 (±0.27) × 10^6^	32	7.9 (±0.38) × 10^4^
N234Y/I257M	4.1 (±1.9) × 10^3^	3.5 (±4.7) × 10^2^	0	1.6 (±0.24) × 10^4^
N234Y/K286E	1.6 (±0.11) × 10^5^	1.1 (±0.22) × 10^4^	0	2.1 (±0.32) × 10^4^
I257M/K286E	9.8 (±0.29) × 10^3^	1.2 (±0.17) × 10^4^	0	1.0 (±0.10) × 10^4^
N234Y/I257M/K286E	4.3 (±0.49) × 10^6^	4.7 (±0.56) × 10^6^	8	7.0 (±0.37) × 10^4^
				
NA_A*_	6.9 (±1.4) × 10^5^	6.7 (±1.5) × 10^5^	2	1.7 (±0.26) × 10^3^
				
No NA	1.3 (±0.9) × 10^3^	1.3 (±0.7) × 10^2^	0	4.3 (±0.1) × 10^2^
NA_A_	1.0 (±0.57) × 10^4^	4.9 (±3.4) × 10^2^	0	6.0 (±0.27) × 10^2^
V119E	5.4 (±0.6) × 10^3^	7.0 (±6.6) × 10^2^	0	6.7 (±0.31) × 10^2^
Y234N	1.3 (±0.2) × 10^4^	7.3 (±4.9) × 10^2^	0	4.2 (±0.27) × 10^2^
R241M	1.2 (±0.2) × 10^4^	9.7 (±5.1) × 10^2^	0	4.4 (±0.12) × 10^2^
R345G	1.3 (±0.1) × 10^4^	7.8 (±2.2) × 10^2^	0	4.3 (±0.42) × 10^2^
V119E/Y234N	9.1 (±2.9) × 10^3^	6.9 (±3.9) × 10^2^	0	4.6 (±0.14) × 10^2^
V119E/R241M	1.1 (±0.3) × 10^4^	5.7 (±1.3) × 10^2^	0	4.0 (±0.04) × 10^2^
V119E/R345G	1.2 (±0.1) × 10^4^	5.0 (±0.6) × 10^2^	0	4.2 (±0.16) × 10^2^
Y234N/R241M	7.5 (±3.0) × 10^3^	1.5 (±1.0) × 10^3^	0	4.2 (±0.16) × 10^2^
Y234N/R345G	8.2 (±2.8) × 10^3^	1.1 (±0.9) × 10^3^	0	4.3 (±0.10) × 10^2^
R241M/R345G	1.8 (±0.2) × 10^4^	1.7 (±0.8) × 10^3^	0	8.2 (±0.47) × 10^2^
V119E/Y234N/R241M	1.0 (±0.05) × 10^4^	2.8 (±1.2) × 10^2^	0	4.2 (±0.23) × 10^2^
V119E/Y234N/R345G	1.0 (±0.3) × 10^4^	3.3 (±1.8) × 10^2^	0	4.4 (±0.29) × 10^2^
V119E/R241M/R345G	2.1 (±0.25) × 10^5^	1.1 (±0.1) × 10^4^	0	5.4 (±0.09) × 10^2^
Y234N/R241M/R345G	ND	ND	ND	ND
V119E/Y234N/R241M/R345G	2.5 (±0.6) × 10^6^	1.5 (±0.1) × 10^6^	2	2.7 (±0.99) × 10^3^
				
NA_C _(NA_A _176-230)	1.6 (±0.05) × 10^7^	3.0 (±0.57) × 10^6^	64	5.5 (±0.08) × 10^4^
NA_C _(NA_A _88-230)	5.0 (±0.50) × 10^5^	1.9 (±0.58) × 10^4^	0	2.3 (±0.07) × 10^4^
NA_C _(NA_A _1-230)	1.6 (±0.39) × 10^5^	6.8 (±2.1) × 10^3^	0	4.5 (±0.10) × 10^3^
S95R	4.3 (±0.07) × 10^5^	1.9 (±0.5) × 10^4^	0	2.1 (±0.12) × 10^4^
V99I/H100Y	5.9 (±0.6) × 10^5^	2.6 (±0.1) × 10^4^	0	3.1 (±0.03) × 10^4^
V119E	1.4 (±0.08) × 10^7^	2.1 (±0.1) × 10^6^	16	3.0 (±0.09) × 10^4^
N146S	1.5 (±0.2) × 10^5^	7.0 (±1.5) × 10^3^	0	1.1 (±0.02) × 10^4^
H155Y/T157A	3.8 (±0.8) × 10^5^	2.4 (±0.3) × 10^4^	0	2.0 (±0.06) × 10^4^
				
NA_C _(NA_A _226-470)	1.3 (±0.35) × 10^4^	1.1 (±0.5) × 10^3^	0	4.0 (±0.09) × 10^2^
NA_C _(NA_A _226-362)	1.1 (±0.50) × 10^4^	1.4 (±0.5) × 10^4^	0	4.3 (±0.02) × 10^2^
NA_C _(NA_A _226-301)	1.2 (±0.09) × 10^5^	2.5 (±0.7) × 10^3^	0	1.3 (±0.02) × 10^3^
Y234N	7.2 (±0.56) × 10^6^	3.6 (±0.3) × 10^5^	32	1.5 (±0.04) × 10^4^
N247D/W248G/Q249L	1.0 (±0.31) × 10^6^	6.2 (±2.6) × 10^3^	0	1.5 (±0.09) × 10^3^
M257I	7.1 (±1.37) × 10^6^	6.0 (±1.0) × 10^4^	4	1.2 (±0.01) × 10^4^
V263T/V266I	6.5 (±0.80) × 10^4^	1.1 (±0.6) × 10^3^	0	4.8 (±0.37) × 10^2^
E269N	2.5 (±0.52) × 10^5^	6.4 (±3.0) × 10^3^	0	1.1 (±0.07) × 10^3^
Y273S	1.2 (±0.07) × 10^5^	2.9 (±0.6) × 10^3^	0	5.0 (±0.26) × 10^2^
A284T	1.0 (±0.08) × 10^5^	2.6 (±1.0) × 10^3^	0	6.2 (±0.15) × 10^2^
E286K	1.8 (±0.004) × 10^7^	2.2 (±0.1) × 10^6^	Und	1.6 (±0.04) × 10^4^
I287V	6.1 (±0.45) × 10^5^	1.2 (±0.1) × 10^4^	0	1.2 (±0.01) × 10^3^
T288M	1.6 (±0.06) × 10^6^	6.0 (±0.3) × 10^4^	0	5.2 (±0.18) × 10^3^

^α ^All values are in relative light units (RLU). Standard errors of the means are in parentheses.

ND, not determined

Und, undiluted

**Figure 3:**
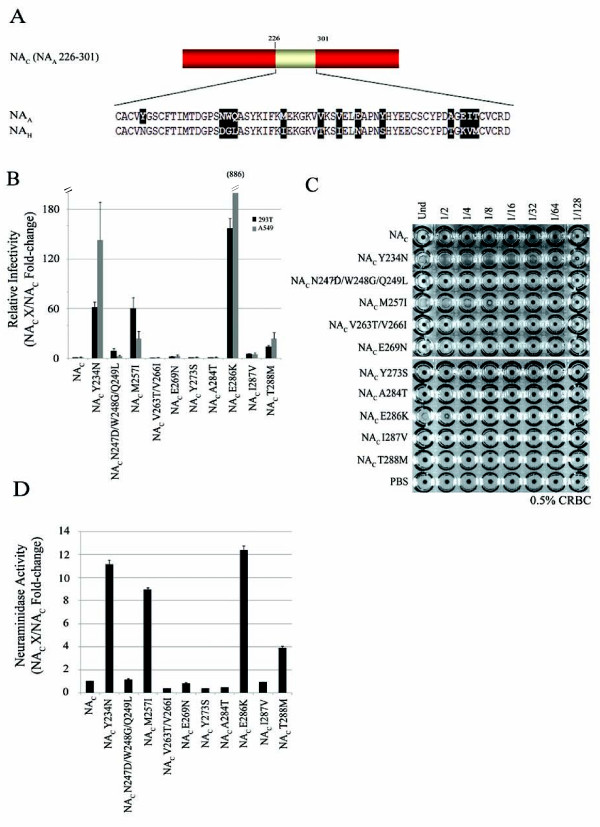
**Molecular analysis of amino acid region 226-301 in NA function**. *A*, schematic of NA_C _(NA_A _226-301). NA_H _sequence is colored red and NA_A _sequence is colored yellow. Single and combined substitutions generated in NA_C _(NA_A _226-301) are shaded. *B*, relative infectivity of 293T and A549 target cells challenged with pseudovirus produced in the presence of respective NA_C _(NA_A _226-301) mutants. Luciferase activity is presented as fold-change. Experiments were performed in triplicates and repeated several times, and error bars indicate standard deviations. *C*, hemagglutination activity of NA_C _(NA_A _226-301) substitution panel. Pseudovirions were mixed with chicken red blood cells and HA titers recorded. *D*, neuraminidase activity of NA_C _(NA_A _226-301) substitution mutants measured by the release of fluorogenic substrate 4-MUNANA. Experiments were performed in triplicates and repeated several times, and error bars indicate standard deviations.

To identify the critical residue(s) for NA function, we took a gain-of-function approach and systematically substituted the variable residues within aa regions 88-176 (Table 1) and 226-301 of NA_C _(NA_A _88-230) and NA_C _(NA_A _226-301), respectively (Figure 3A and Table 1). For example, tyrosine 234 in NA_C _(NA_A _226-301) was substituted to an asparagine (NA_C _Y234N), the existing NA_H _residue corresponding at this position. For NA_C _(NA_A _226-301), a total of ten, either single or combined, substitutions were generated (Figure 3A). Three substitutions introduced within NA_C _(NA_A _226-301), Y234N, M257I, and E286K, resulted in increased luciferase activity, hemagglutination and NA enzyme activity, as compared to NA_C _(NA_A _226-301) (Figure 3B-D). Y234N gave 32 HA units/ml, M257I resulted in 8 HA units/ml and HA activity for E286K was observed with the undiluted (Und) sample (Figure 3C). These results were generally in agreement with the enzymatic data. However, we did not observe a strong correlation between the magnitude of the HA and the magnitude of the infectivity as measured by the luciferase assay. For example, mutant Y234N displayed higher HA activity than E286K (32 *vs *undiluted), but it gave a lower level of infectivity than that of E286 (also see Table 1). The nature of this discrepancy needs to be further examined in the future.

The NA_C _(NA_A _226-301) constructs with Y234N, M257I, and E286K substitutions resulted in increased levels of NA activity (Figure 3D). Taken together, these results indicate that N234, I257, and K286 of NA_H _are critical for NA activity and hence function in pseudoviral particle release. Using the same approach, we also demonstrated a critical role of E119 within NA_C _(NA_A _88-230) for enzyme activity (Table 1).

**Figure 4:**
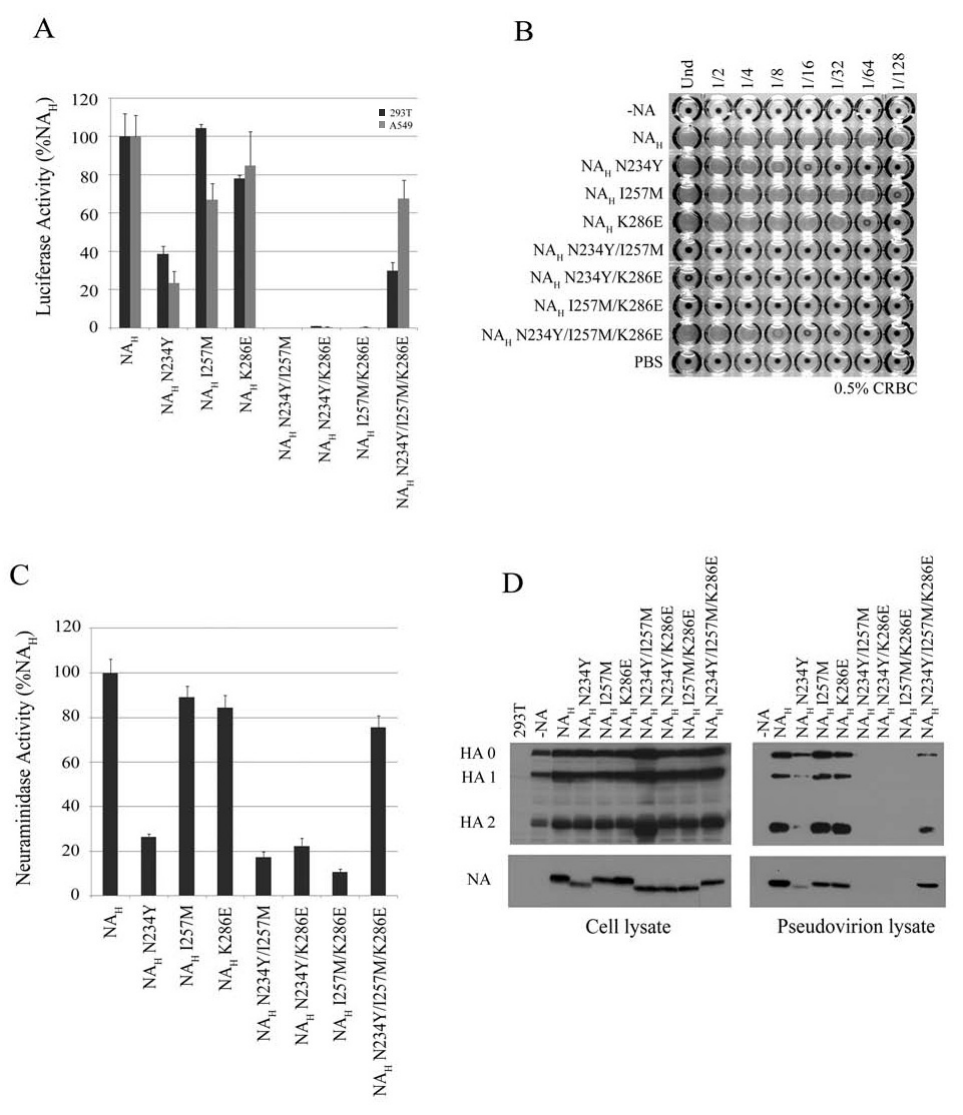
**Characterization of critical residues necessary for NA function**. *A*, relative infectivites of pseudovirions produced in the presence of NA_H _N234Y, I257M and K286E single and combined substitution mutants (N2 numbering). Experiments were performed in triplicates and repeated several times, and error bars indicate standard deviations. *B*, hemagglutination activity of NA_H _substitution panel. *C*, neuraminidase activity of NA_H _substitutions measured by the release of fluorogenic substrate 4-MUNANA. Enzyme activity is presented as the fold-change of triplicate samples. Experiments were performed in triplicates and repeated several times, and error bars indicate standard deviations. *D*, western blot of HA and NA expression in 293T cell lysates and incorporation onto HIV particles. The HA precursor (HA0), and proteolytic subunits (HA1 and HA2) are detected in both cell and virus lysates.

To further examine the potential role of these residues of NA, N234Y, I257M, and K286E substitutions, single or combined, were introduced into NA_H_. The single NA_H _substitutions marginally effected NA function. NA_H _double substitution mutants, N234Y/I257M, N234Y/K286E and I257M/K286E, completely abolished NA activity (Figure 4A-C, and Table 1). Interestingly, NA_H _triple substitution mutant resulted in a revertant phenotype. Its enzymatic activity was comparable to NA_H_, and it displayed restored luciferase levels and partially restored hemagglutination (Figure 4 and Table 1).

All NA mutants were expressed in the producer cells. NA_H _N234Y and all three double substitution mutants migrated faster than NA_H _on the SDS-PAGE gel (Figure 4D, left panel). the double substitution NA mutants were not expressed on pseudoviral particles (Figure 4D, right panel). The triple NA mutant showed a similar migration pattern to NA_H _and it was expressed on pseudovirions (Figure 4D, right panel). These data correlated with the NA_H _triple mutant restored activity.

### Identification of critical residues for NA function

**Figure 5:**
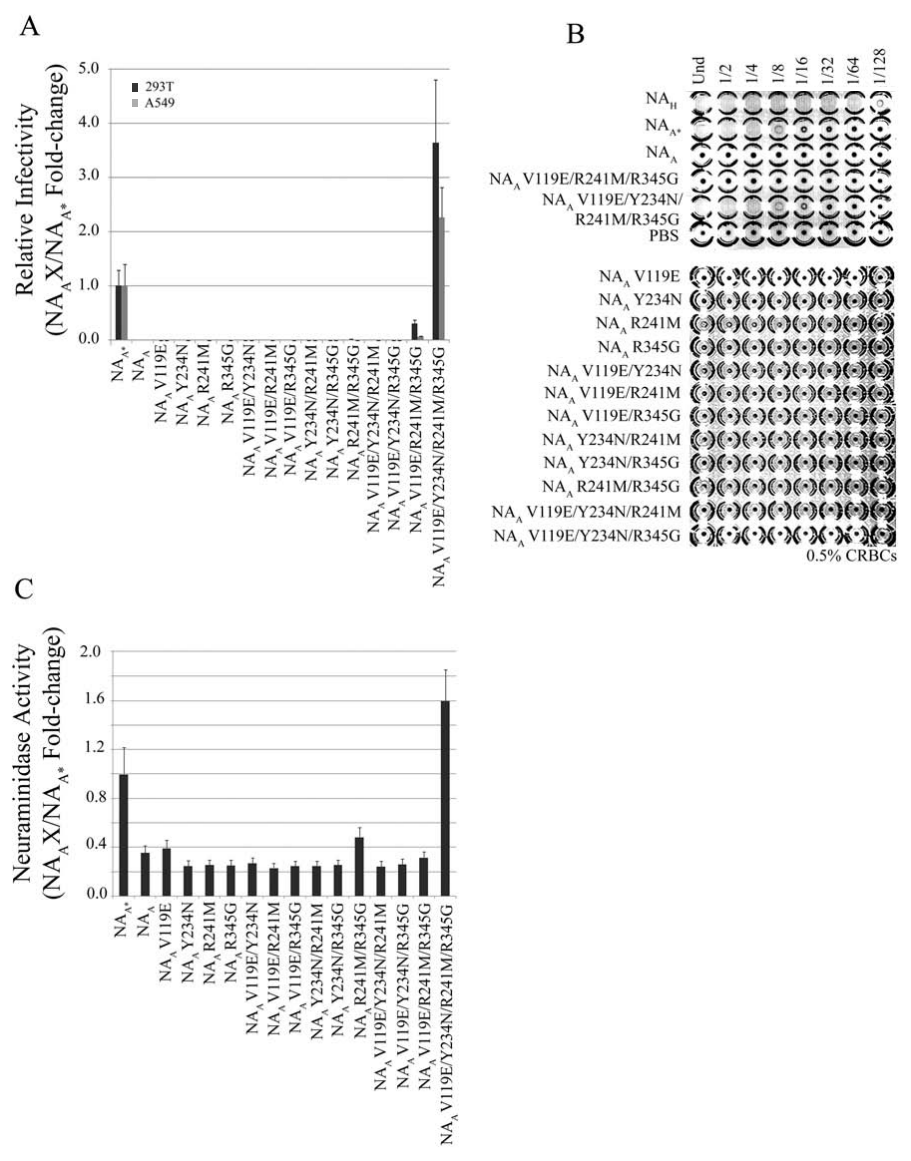
**NA_A _gain-of-function in pseudoviral particle release**. *A*, luciferase activity of pseudovirions generated in the presence of NA_A _and NA_A _mutants containing single or combined substitutions V119E, Y234N, R241M and R345G (N2 numbering). Experiments were performed in triplicates and repeated several times, and error bars indicate standard deviations. *B*, hemagglutination activity of NA_A _substitution panel. *C*, neuraminidase activity of NA_C _(Av 226-301) substitution mutants measured by the release of fluorogenic substrate 4-MUNANA. Experiments were performed in triplicates and repeated several times, and error bars indicate standard deviations.

An NA_A _variant derived from the A/chicken/Henan/2004 (H5N1) mRNA, henceforth referred to as NA_A*_, was generated and displayed an intermediate phenotype in NA function. NA_A* _resulted in a hemagglutination titer of 8 HA units/ml and enhanced enzyme activity compared to NA_A _(Figure 5B, Table 1). Sequence alignment indicated that NA_A* _contains six amino acid differences from NA_A_. Isoleucine at position 8 of NA_A* _is located in the transmembrane domain, and differs from Thr8 of NA_A_. The remaining five NA_A* _residues, E119, N234, M241, G248 and G345, which are conserved with NA_H _at the corresponding positions, but differ from that of NA_A _(V119, Y234, R241, W248 and R345) are located in the ectodomain of the protein.

**Figure 6:**
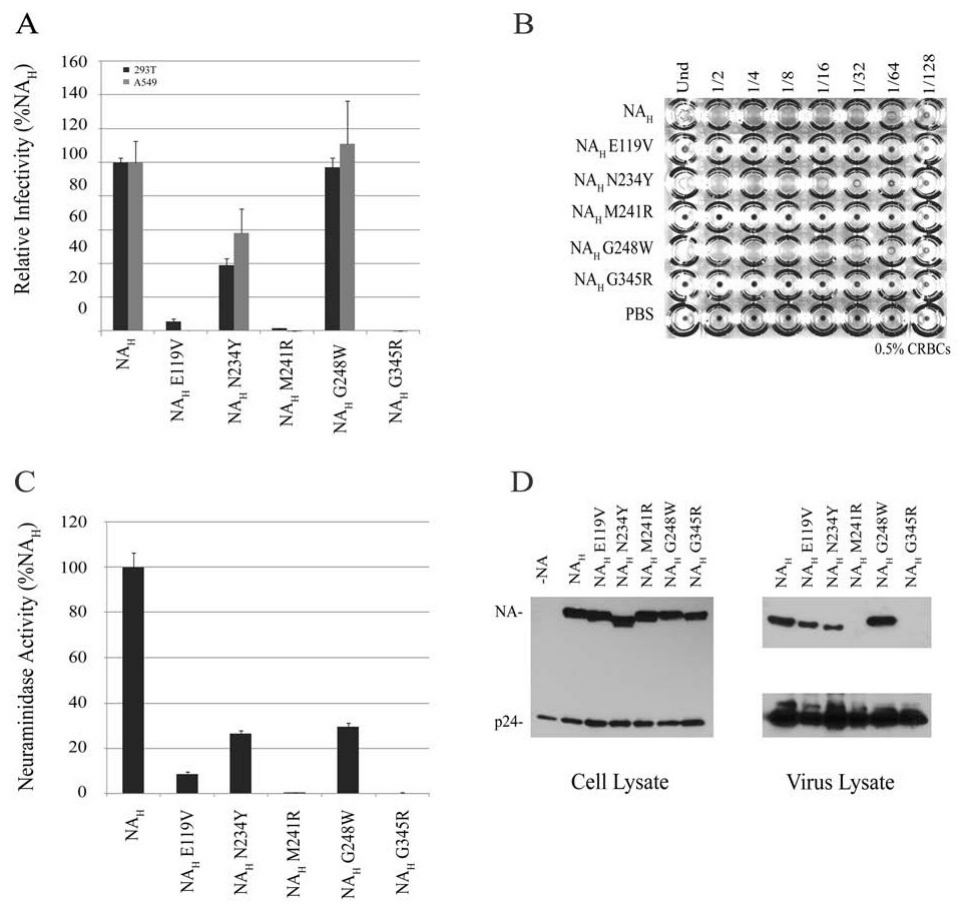
**Effect of E119V, N234Y, M241R and G345R single substitutions on NA_H _function**. *A*, luciferase activity of pseudovirions generated in the presence of NA_H _single substitution mutants. Experiments were performed in triplicates and repeated several times, and error bars indicate standard deviations. *B*, hemagglutination activity of NA_H _substitution panel. *C*, neuraminidase activity of NA_H _substitution mutants measured by the release of fluorogenic substrate 4-MUNANA. Values are presented as the fold-change of triplicate samples. Experiments were performed in triplicates and repeated several times, and error bars indicate standard deviations. *D*, western blot of NA expression in 293T cells and incorporation onto HIV particles.

To examine the potential roles of these five residues, we first took a gain-of-function approach to determine which amino acid substitutions were required to restore NA_A _function. Since NA_H _substitution mutant G248W was comparable to NA_H _(see Table 1), we focused on the following residues of NA_A_, V119, Y234, R241 and R345. As shown in Figure 6, all four substitutions in combination were required to restore NA_A _to a level comparable to NA_A*_. These results demonstrated that these four residues are critical in maintaining NA activity.

Second, a loss-of-function approach was used to determine which amino acid substitutions adversely affected the function of NA_H_. Substitutions E119V, M241R and G345R abolished NA_H _activity whereas substitution N234Y resulted in a moderate reduction in NA enzyme activity and luciferase levels from target cells. In contrast, substitution G248W did not adversely affect the NA-mediated HIV pseudovirus release (Figure 6A and 6C, and Table 1). In general, these results were consistent with that of the HA assay (Figure 6B and Table 1). NA_H _substitution mutants M241R, G345R and E119V resulted in no hemagglutinating virions, while the HA titer of NA_H _G248W was comparable to parental NA_H _and NA_H _N234Y resulted in 16 HA units/ml. Thus, these results indicate that two additional residues (M241 and G345) are also critical for NA_H _activity. All NA mutants were expressed in producer cell lysates and in pseudovirion lysates with the exception of NA_H _mutants, M241R and G345R (Figure 6D). To ensure that the mutations did not adversely affect NA folding or transport, we evaluated NA cellular surface expression using a biotinylation assay. It was found that NA_H _single and combined substitutions did not disrupt the capacity for NA to be properly folded and transported to the plasma membrane. All NA mutants in this panel were detected on the surface and showed comparable expression levels (data not shown). Hence, the defective NA function observed with NA_H _double substitution mutants and NA_H _M241R and G345R single mutants is not a result of reduced cell surface expression levels.

## Discussion

**Figure 7:**
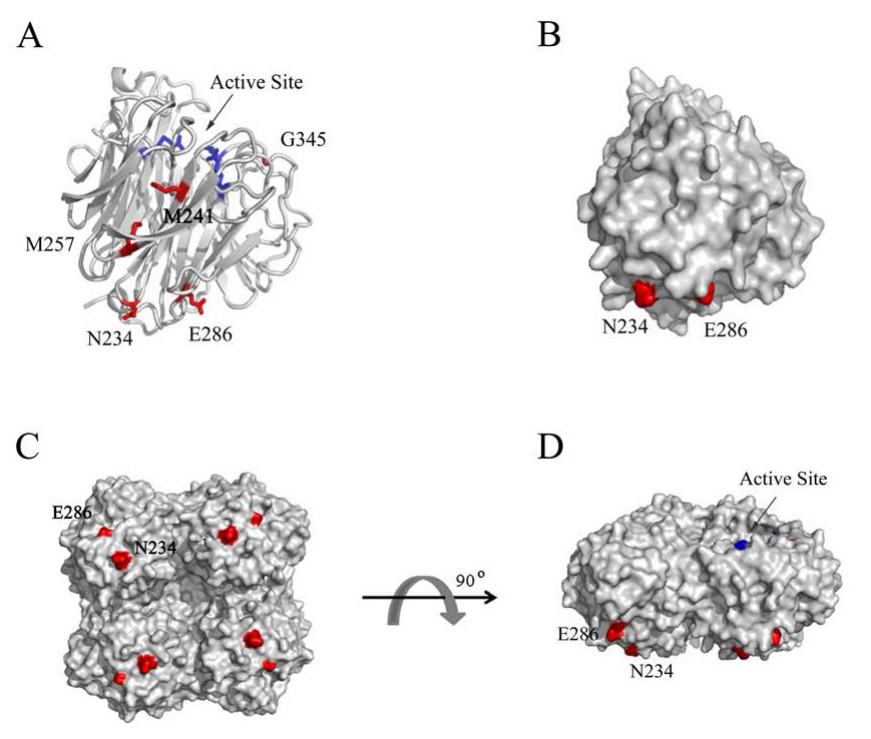
**Projection of critical NA residues onto the tertiary structure**. *A*, NA monomer ribbon diagram. Tri-arginyl residues (R118, R292 and R371) are labeled blue for reference of the enzyme active site. Residue substitution sites (N234, M241, M257, E286, and G345) are labeled red. *B*, NA monomer surface representation. N234 and E286 are shown in red. *C*, NA tetramer surface representation. View is looking up from the virion surface at the underside of the globular head. N234 and E286 are shown in red. *D*, side profile view of NA tetramer surface representation, parallel to the viral membrane. N234 and E286 are labeled red and the active site is labeled blue. The NA structures are displayed using PyMOL published by DeLano Scientific. PDB ID: 2HTY[34,35].

In this study, we sought to identify important residues for influenza virus NA function. Previous reports have identified numerous critical residues forming the NA enzyme active site and surrounding framework (in N2 numbering): Arg 118, Glu 119, Asp 151, Arg 152, Asp 198, Ile 222, Arg 224, Glu 227, Asp 243, His 274, Glu 276, Glu 277, Arg 292, and Asp 330, Arg 371 [19,25-28]. The goal of the current work was to further examine and identify additional critical residues of NA using an established pseudotyping system to study influenza virus envelope glycoprotein function [24]. We demonstrated HIV/HA pseudovirion production may be used as a suitable surrogate system to indirectly measure viral particle release from 293T producer cells. Through an extensive molecular analysis, we identified and characterized critical molecular determinants at the following five positions: 234, 241, 257, 286 and 345 In mapping these positions onto the three-dimensional structure of avian H5N1 NA, we found that these residues, with the exception of G345, are distal to the active site (Figure 7). Internal residues, M241 and M257, are part of β-sheet 3, while residues 234 and 286 are surface exposed and localize more closely together on the ectodomain surface proximal to the virion membrane. G345 is part of an antigenic site (aa 339-347) located on the rim of the enzyme pocket. Sequence alignment of over 8,000 NAs revealed greatest amino acid variability at positions 257 and 286 and to a lesser extent at position 234. In contrast, M241 and G345 are highly conserved with a very low frequency of amino acid variations observed at these two positions.

This study was initiated when it was observed that two NA genes demonstrated distinct phenotypes. An NA gene derived from mouse-adapted PR8 influenza virus (NA_H_) could efficiently facilitate release of HIV/HA pseudovirions from the producer cells, whereas an avian H5N1 NA clone (NA_A_) was completely defective. By creating a panel of chimeric constructs between these two genes, we were able to identify two regions of NA, aa regions 88-176 and 226-301, likely responsible for the functional difference of these two NAs (see Figure 2). Mutational analysis of these regions led to identification of critical molecular determinants at positions 234, 257 and 286. We also identified residues 241 and 345, and further confirmed the importance of residue 234 by restoring NA_A _activity. Introduction of arginine at either position 241 or 345 completely abrogated NA function; however, NA_H _M241R and G345R mutants were detected on the cells surface through biotinylation. Hence, the defective NA function observed with NA_H _M241R and G345R single mutants is not a result of reduced expression on the cell surface.

Viral envelope modifications, including glycosylation patterns, may dictate, in part, viral glycoprotein function. The asparagine residue found at position 234 is surface exposed (see Figure 7) and is part of a conserved motif (Asn-X-Ser/Thr) that denotes an N-linked glycosylation site. NAs containing N234Y substitution displayed a mobility shift on an SDS-PAGE gel suggesting that elimination of this glycosylation site on NA resulted in the loss of the carbohydrate modification. Interestingly, a similar mobility shift was also observed with the NA_H _I257M/K286E double substitution mutant that retained N234. We speculate that this was likely due to a misfolded NA which altered or masked the glycosylation site and prevented carbohydrate attachment. Glycosylation of N234 may be important for NA activity required to mediate the release of HA-containing HIV particles; however, like all the NA mutants, the mutation does not affect protein expression on the cellular surface.

It is interesting to point out that the individual substitutions N234, I257 or K286 in NA_H _impaired NA function, but a more striking defect was observed when any two substitutions were combined (see Figure 4). All three double mutants were detectable in the cell lysates, with a faster migrating position on SDS-PAGE, but these mutants were not detected on the cell surface explaining their absence on the HIV pseudovirions. However, the triple mutant behaved more like NA_H_, with greatly restored NA activities. Further analysis of the roles of these residues may provide insights on NA structure and function. Since many of these residues are clustered on the underside of the large globular head when projected onto the three-dimensional structure of avian influenza H5N1 NA. We speculate that this region may be a unique target site on NA for the design of novel inhibitors.

Currently, NA inhibitor oseltamivir carboxylate (Tamiflu) is the best available drug for influenza treatment. Its efficacy, however, is limited due its high propensity to select for drug-resistant variants, which is becoming an increasing problem with seasonal influenza strains. Thus, it is important to continue efforts for the design and development of more potent and efficacious drugs to treat influenza infection. The region in which residues 234, 286-both of which are surface exposed and lie adjacent to each other on the underside of the ectodomain-and internal residue 257 may offer a unique opportunity to explore a new target region on NA, particularly since we have demonstrated this region's importance in NA function. We believe that these assays can be adapted for characterizing the NA function of 1918 pandemic influenza virus to alleviate safety concerns. Of interest, residue 234 of H1N1 NA has been recently demonstrated to be a "permissible" secondary mutation site which may allow the virus to compensate for the reduced fitness of oseltamivir resistance phenotype of H274Y[29], underlying the importance of the residue 234 identified as being important in NA function.

## Methods

### Cell lines

Human 293T cells were maintained in Dulbecco's modified Eagle's Medium (DMEM; HyClone) supplemented with 10% fetal bovine serum (FBS; HyClone), 100 μg/ml of streptomycin and 100 units of penicillin (Invitrogen). Human lung epithelial A549 cells were grown in Roswell Park Memorial Institute medium (RPMI; Invitrogen) supplemented with 10% fetal bovine serum (FBS; HyClone), 100 μg/ml of streptomycin, 100 units of penicillin (Invitrogen), 1% Na-pyruvate and 1% 1 M HEPES.

### Constructs and reagents

The HA gene is from highly pathogenic avian influenza A/Goose/Qinghai/59/2005 (H5N1) virus isolated from infected migratory waterfowl found in Lake Qinghaihu, Qinghai Province, in western China [30]. NA cDNA from the mouse-adapted A/Puerto Rico/8/1934 (H1N1; PR8) influenza virus strain was kindly provided by John Olsen, University of North Carolina [31]. The mRNA and plasmid borne NA gene were derived from highly pathogenic avian influenza A/chicken/Henan/12/2004 (H5N1) virus. The HIV-1 vector pNL4-3.Luc.R^-^E^- ^and mouse monoclonal anti-p24 HIV antibody were obtained through the NIH AIDS Research and Reference Reagent program (Germantown, MD). Polyclonal anti-HA (H5) NR163 was obtained from the Biodefense and Emerging Infections Research Resources Repository (Manassas, VA).

NA chimeric constructs were created by a two-step PCR technique with custom designed, overlapping primers. The amplified fragments were cloned into pEF6/V5-His-TOPO vector (Invitrogen) using KpnI and NotI restriction enzymes and the sequence was confirmed by DNA sequencing. Influenza virus mRNA from A/chicken/Henan/12/2004 (H5N1) was reverse transcribed into cDNA using primer set (5'to 3'acacggagcaaaagcagg and acgcggagtagaaacaagg) [32]. The NA cDNA was amplified using segment-specific primers and the resulting PCR product inserted into pEF6/V5-His-TOPO vector (Invitrogen). Site-directed mutagenesis of the NA genes was performed using the Strategene Quick-Change mutagenesis kit following the supplier's protocol with custom designed primers carrying the designated mutation. All NA mutants were confirmed by DNA sequencing of the full-length NA gene.

### Production of pseudovirions and infection assay

Influenza pseudovirions were produced in 293T cells using a polyethylenimine (PEI)-based transfection protocol. Plasmid DNAs were co-transfected into 293T cells and after 48 h post-transfection, culture supernatants were collected, filtered through a 0.45 μm-pore size filter (Nalgene) and used to directly infect 293T and A549 target cells. Empty vector was used in place of omitted plasmids where appropriate. Relative infectivity was determined 48 h post-infection by measuring luciferase activity of the reporter gene using an FB12 luminometer at 10 second intervals (Berthold detection system). Each sample was done in triplicate and the experiments repeated at least three times. For NA treatment, a commercial neuraminidase (*Clostridium perfringens; *New England Biolabs) was added to producer cells at 26 h and 46 h post-transfection at a concentration of 5 units/ml.

### Hemagglutination assay

Producer cell culture supernatants were harvested 48 h post-transfection, filter-sterilized and concentrated over a 30% Sucrose-NTE cushion consisting of 100 mM NaCl, 10 mM Tris (pH 7.4), and 1 mM EDTA by centrifugation at 55,000 rpm for 2 h in a SW55Ti rotor at 4°C. Pseudovirion pellets were resuspended in 50 μl of Tris-buffer containing 50 mM Tris-HCl (pH 7.5), 150 mM NaCl, and 5 mM EDTA. Twofold serial dilutions were mixed with an equal volume of 0.5% chicken erythrocyte suspension (CRBCs; Lampire Biological Laboratories) and incubated in a U-bottomed 96-well plate at 4°C. HA titers were recorded after 1 h. Hemagglutination assay experiments were repeated at least three times.

### Neuraminidase enzyme assay

A standard fluorometric enzyme assay developed by Potier *et al*. was adapted to measure NA activity [33]. Producer cell lysates transfected with representative NA constructs were added to NA fluorogenic substrate 2'-(4-methylumbelliferyl- *N*-acetylneuraminic acid (4-MUNANA; Sigma) to a final concentration of 100 μM. The reactions were carried out in 50 μl of 33 mM MES (pH 6.5) containing 4 mM CaCl_2 _in 96-well black Opti-plates (BD Biosciences) and incubated in a 37°C water bath for 1 h. The reactions were terminated by adding 150 μl stop solution containing 0.5 M NaOH (pH 10.7) and 25% ethanol. The fluorescence of released 4-methylumbelliferone was measured using a Labsystems Fluoroskan II spectrophotometer (PerkinElmer). The excitation wavelength was set at 355 nm, and the emission wavelength was set at 460 nm. Samples were done in triplicate and the experiments repeated at least three times.

### Immunoblot analysis

NA genes modified to express a C-terminal HA-epitope tag were used to analyze NA protein expression in the producer cell lysates and NA incorporation onto HIV particles. 293T cells were transfected with representative NA plasmids and 48 h post-transfection the cells were washed in ice-cold PBS and then lysed in 0.2 ml of Triton X-100 lysis buffer containing 50 mM Tris-HCl (pH 7.5), 150 mM NaCl, 5 mM EDTA, 1% Triton-X, and a protease inhibitor cocktail. The cell culture supernatants were layered over a 20% sucrose cushion and centrifuged at 55,000 rpm for 45 min in a SW55Ti rotor at 16°C. The pseudovirion pellets were lysed in 1% Triton X-100 lysis buffer, samples subjected to SDS-PAGE and then transferred to a PVDF membrane. NA was detected with monoclonal anti-HA antibody (Sigma) and HIV core with monoclonal anti-p24 antibody. The membrane was then probed with peroxidase-conjugated goat anti-mouse secondary antibody and the protein bands visualized by chemiluminescence (Pierce).

### NA cellular surface expression

Producer cells were transfected with representative NA plasmids and 48 h post-transfection the cells were washed and then released in PBS (pH8.0) containing 0.5 mM EDTA. Cell surface proteins were biotinylated for 30 min at 4°C using EZ-Link™ Sulfo-NHS-LC-Biotin reagent (Thermo Fischer Scientific). Cells were washed twice with ice-cold PBS (pH 8.0) containing 100 mM glycine, once with ice-cold PBS (pH 8.0), and then lysed in Triton-X 100 lysis buffer containing a protease inhibitor cocktail. Biotinylated surface proteins were separated from cell lysates using NeutrAvidin Agarose Resin (Thermo Fisher Scientific) by incubating mixtures overnight at 4°C. Agarose beads were washed twice with PBS (pH 8.0) and resuspended in PBS. Samples were subjected to SDS-PAGE analysis and NA proteins were detected following the immunoblot protocol described above.

## Competing interests

The authors declare that they have no competing interests.

## Authors' contributions

JRT, YG, KC, JY, JW, YC, and LR participated in the study design, JRT, YG, KC, JY performed the experiments, and all authors participated in manuscript writing and revising. All authors read and approved the final manuscript.
